# Ultrasound Shear Wave Elastography and Transient Optical Coherence Elastography: Side-by-Side Comparison of Repeatability and Accuracy

**DOI:** 10.1109/OJEMB.2021.3075569

**Published:** 2021-04-27

**Authors:** JUSTIN R. RIPPY, MANMOHAN SINGH, SALAVAT R. AGLYAMOV, KIRILL V. LARIN

**Affiliations:** University of Houston, Houston, TX 77204 USA

**Keywords:** Biomedical optical imaging, elastography, phantoms, shear wave, ultrasound elastography

## Abstract

**Objective:**

We compare the repeatability and accuracy of ultrasound shear wave elastography (USE) and transient optical coherence elastography (OCE).

**Methods:**

Elastic wave speed in gelatin phantoms and chicken breast was measured with USE and OCE and compared with uniaxial mechanical compression testing. Intra- and Inter-repeatability were analyzed using Bland-Altman plots and intraclass correlation coefficients (ICC).

**Results:**

OCE and USE differed from uniaxial testing by a mean absolute percent error of 8.92% and 16.9%, respectively, across eight phantoms of varying stiffness. Upper and lower limits of agreement for intrasample repeatability for USE and OCE were ±0.075 m/s and −0.14 m/s and 0.13 m/s, respectively. OCE and USE both had ICCs of 0.9991. In chicken breast, ICC for USE was 0.9385 and for OCE was 0.9924.

**Conclusion:**

OCE and USE can detect small speed changes and give comparable measurements. These measurements correspond well with uniaxial testing.

## I. INTRODUCTION

The underlying mechanical properties of tissues are important for organ development, cell migration, cell behavior, and wound healing. Because tissue mechanical properties are relevant to many biological processes and disease states, it is essential to be able to measure them with both high precision and accuracy.

Elastography is a well-established noninvasive imaging technique to assess tissue mechanical properties. When performing elastography, the tissue is deformed and the tissue response is measured. Often, dynamic techniques are preferred since they do not require *a priori* knowledge of the excitation forces. The most common dynamic technique is shear wave elastography, where a mechanical wave is induced in tissue and tracked via one of several imaging modalities. The wave speed can then be used to estimate tissue mechanical properties.

Commonly used imaging modalities in elastography are magnetic resonance imaging (MRI) [1], ultrasound (US) [2], and optical coherence tomography (OCT) [3]. MRI is capable of imaging tissues that are not possible with US or OCT, such as the brain [4], [5]. MRI is ubiquitous in clinical settings but is several orders of magnitude higher in cost than either ultrasound or OCT and requires much more space. Additionally, elastography using high-resolution MRI suffers from relatively poor resolution in comparison with ultrasound and OCT, with typical values in the 1–2 mm^3^ range [4]–[6], and requires long imaging times to obtain high contrast of microscale structures. Ultrasound benefits from widespread integration by hospitals over the past several decades, the ability to penetrate deeply into tissues (cm-scale), and with the advent of ultrahigh-frequency ultrasound transducers (100–300 MHz), the ability to obtain both axial and lateral resolutions approaching optical imaging resolutions [7]. However, the majority of ultrasound systems operate at much lower frequencies (1–40 MHz) due to challenges in device fabrication, attenuation, and other artifacts, resulting in typical resolutions in the tens to hundreds of micrometers up to millimeter-scale [7]. Optical coherence elastography (OCE) has been extensively developed for ocular tissues, tumor detection, evaluating skin stiffness, vasculature, and other tissues. OCT has superior axial and lateral resolution compared to ultrasound, with typical values of only a few micrometers. Additionally, it benefits from fast acquisition speed but has limited penetration depth due to light attenuation in tissue (limited to ≤2 mm in highly scattering tissues, such as skin). While either modality can be used for elastography, usually, the choice is determined by experimental constraints and system availability. These constraints include but are not limited to penetration depth required, tissue thickness, and tissue material properties. In many cases, OCE and USE can both be used, such as in the case of skin, eyes, surgically exposed tissues, and excised tissues. At least one study involving both OCE and USE has been performed [8], though more often, ultrasound is used solely as an excitation method [9], [10], or either imaging modality is paired with another modality [11]–[13]. Because either modality may be used to determine tissue properties, a comparison needs to be made between the two to determine what, if any, differences exist in terms of accuracy of the measured value and precision.

**FIG. 1.**
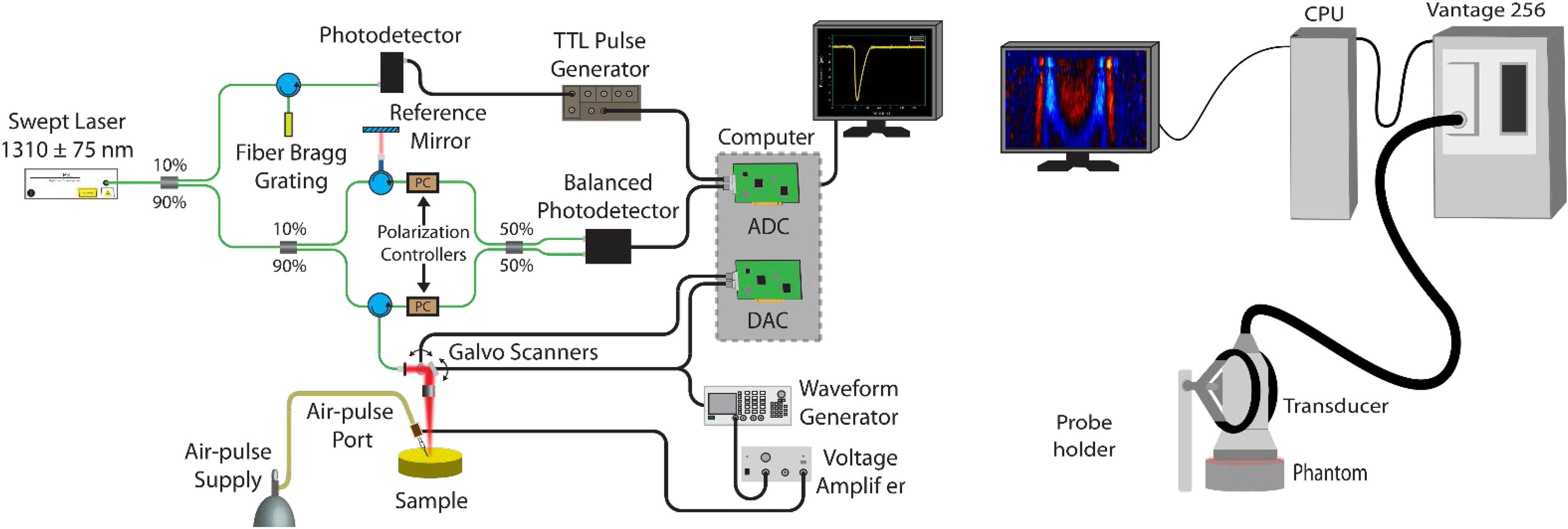
OCE (left) and USE (right) setups. The OCE setup consists of an SSOCT system with coupled air pulse through a blunt needle. The USE system is a Vantage 256 ultrasound system, which uses the probe to perform both excitation and measurement.

In this paper, we directly compare elastography measurements using OCE and USE of the same samples (Fig. 1). We compare wave speed and repeatability on tissue-mimicking gelatin phantoms using transient impulse-based wave elastography and perform Bland-Altman analysis on the results [14]. Additionally, we show comparable results while measuring the mechanical anisotropy of chicken breast muscle.

## II. RESULTS

### A. COMPARISON OF YOUNG’S MODULUS VALUES

**FIG. 2.**
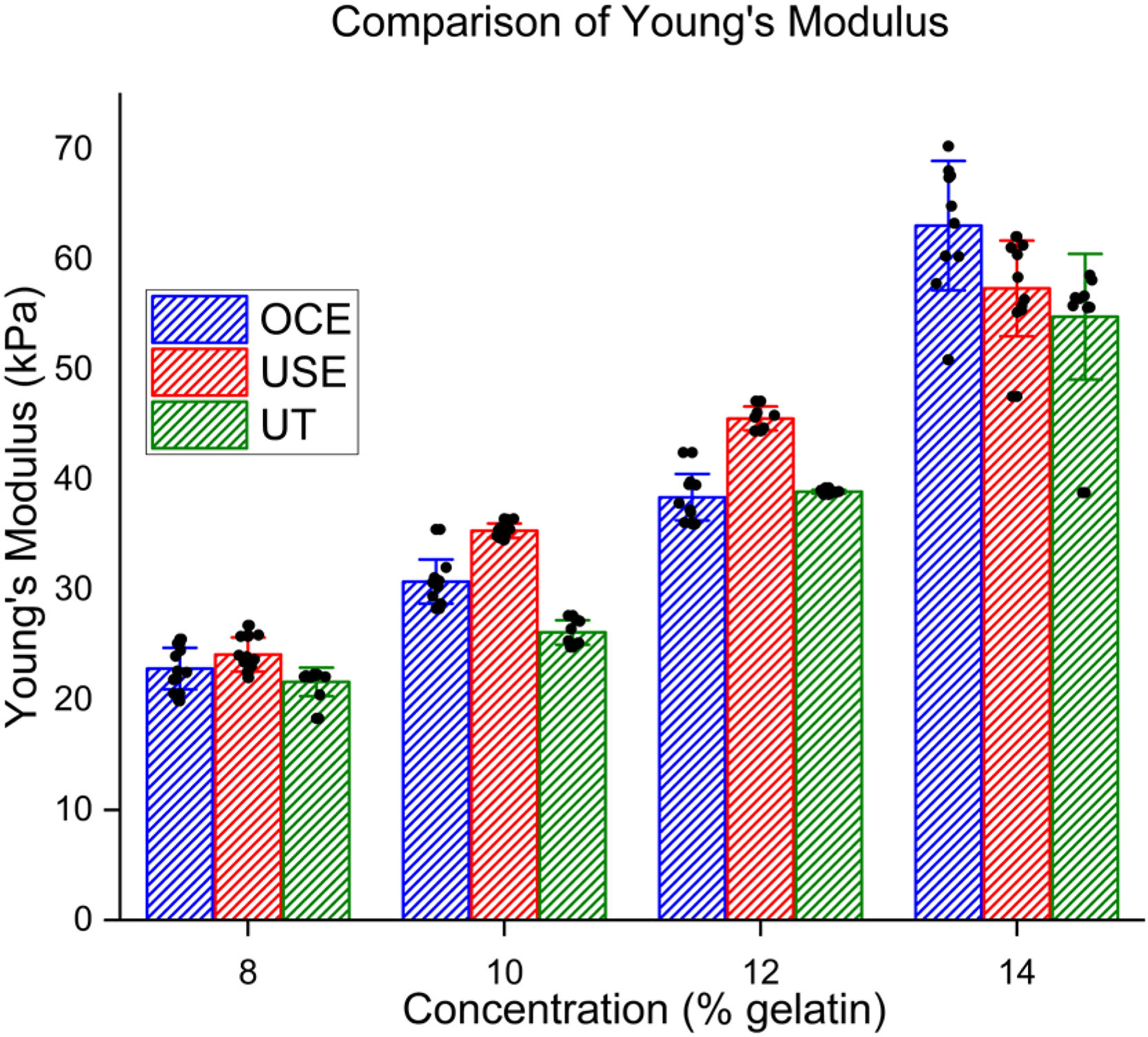
Comparison between Young’s modulus values in gelatin phantoms of various concentrations. USE: Ultrasound elastography. OCE: Optical coherence elastography. UT: Uniaxial testing.

Measured group velocities were used to calculate Young’s modulus and compared with the values obtained via uniaxial testing (UT), as shown in Fig. 2. Numerical results are presented in Table I as intra-concentration averages ± standard deviations (N = 10 measurements for each concentration). For the 8% gelatin phantoms, OCE and USE had errors of 5.6% and 11.4% as compared with UT, respectively. For the 10% phantoms, OCE and USE had errors of 17.7% and 35.4%, respectively. For 12%, OCE and USE had errors of 1.28% and 17.6%, respectively. For 14%, OCE and USE had errors of 11.1% and 3.2%, respectively. USE differed from UT by 3–35%, with a mean absolute percent error of 16.9% across all eight phantoms. OCE differed by 1–18%, with a mean absolute percent error of 8.9% across all eight phantoms.

### B. INTRA- AND INTER-SAMPLE REPEATABILITY

**FIG. 3.**
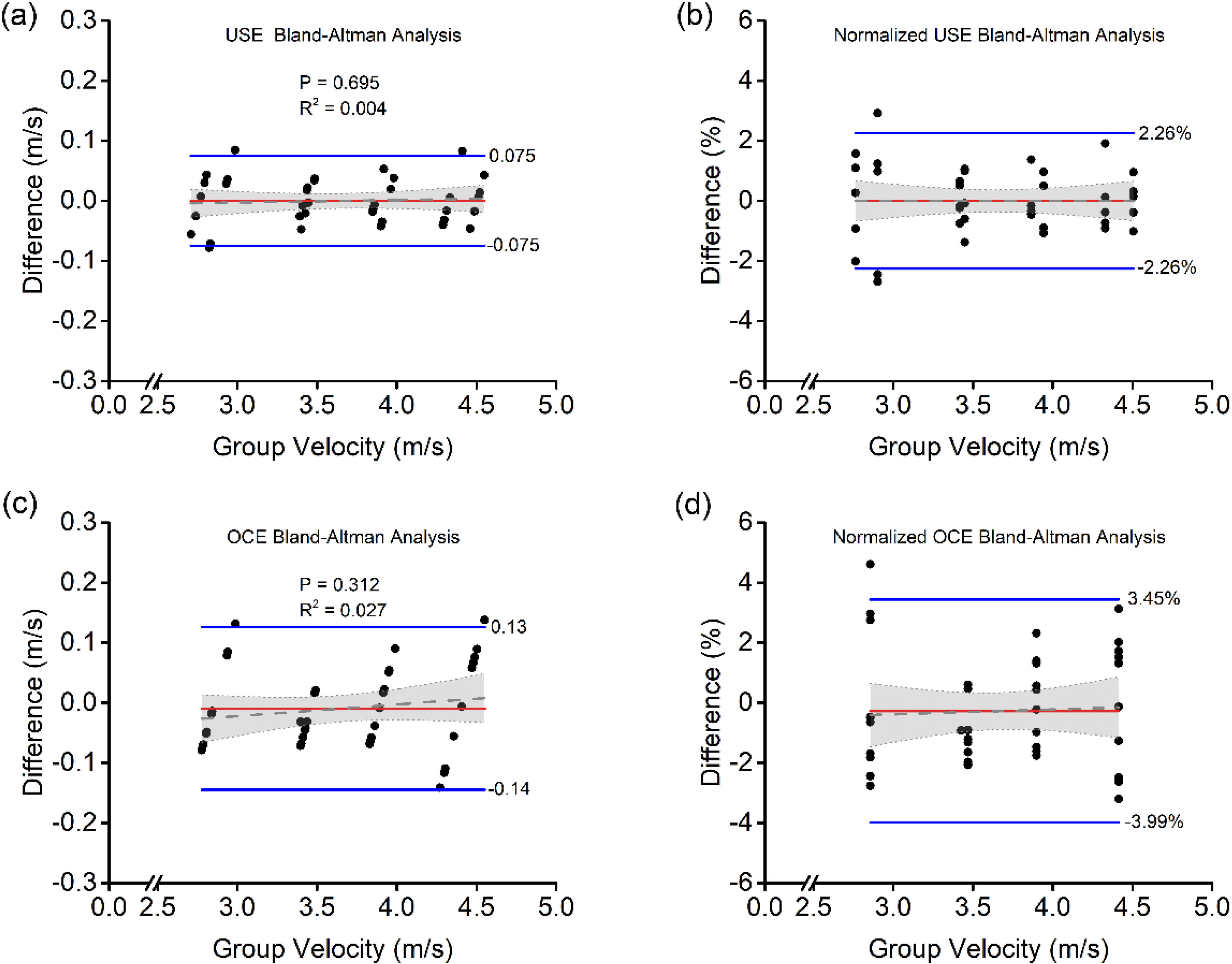
Shear wave group velocity and normalized Bland-Altman plots with corresponding labeled (blue lines) limits of agreement and (red) biases. (a) BA analysis for USE. (b) Normalized BA analysis for USE. (c) BA Analysis for OCE. (d) Normalized BA analysis for OCE.

Fig. 3 shows the Bland-Altman analysis for USE and OCE. Each plot shows the difference in the shear wave velocity of each of the 5 repeated measurements for each phantom compared with the mean speed of the 5 measurements. The spread at each mean speed indicates the variance of intrasample group velocity measurements. Overall, there was no measurable bias from repeated measurements using USE. OCE had a slight bias of −0.009 m/s. The upper and lower limits of agreement for USE were ±0.075 m/s, and the limits of agreement for OCE were −0.14 m/s and 0.13 m/s, as shown in Fig. 3(a) and (c), respectively. The normalized plots in Figures 3b and 3d show that USE had limits of agreement of ±2.26%, while OCE had upper and lower limits of agreement of 3.45% and −3.99%, respectively. Internal repeatability was also analyzed using intraclass correlation coefficients (ICC) [15] (USE: 0.9991, OCE: 0.9991, UT: 0.9994), which are all excellent as expected from phantom measurements.

A linear fit was conducted on the data points on all four Bland-Altman plots to determine if there was any bias with respect to group velocity. For USE, the fit in Fig. 3(a) had a slope of 0.0041 ± 0.010, which was not statistically significant from zero (P=0.695). The slope of the fit of the normalized data in Fig. 3(b) was even closer to zero at 4.38 × 10^−14^ ± 0.31 %/m/s, which was also not significant. For OCE, the fit in Fig. 3(c) had a slope of 0.019 ± 0.02 while the fit of the normalized data in Fig. 3d had a slope of 0.16 ± 0.53 %/m/s, and both were not significant (P=0.312 and 0.761, respectively). These findings indicate that there was no effect on the measurement bias as a function of velocity (i.e., stiffness) in USE or OCE.

### C. CHICKEN BREAST

**FIG. 4.**
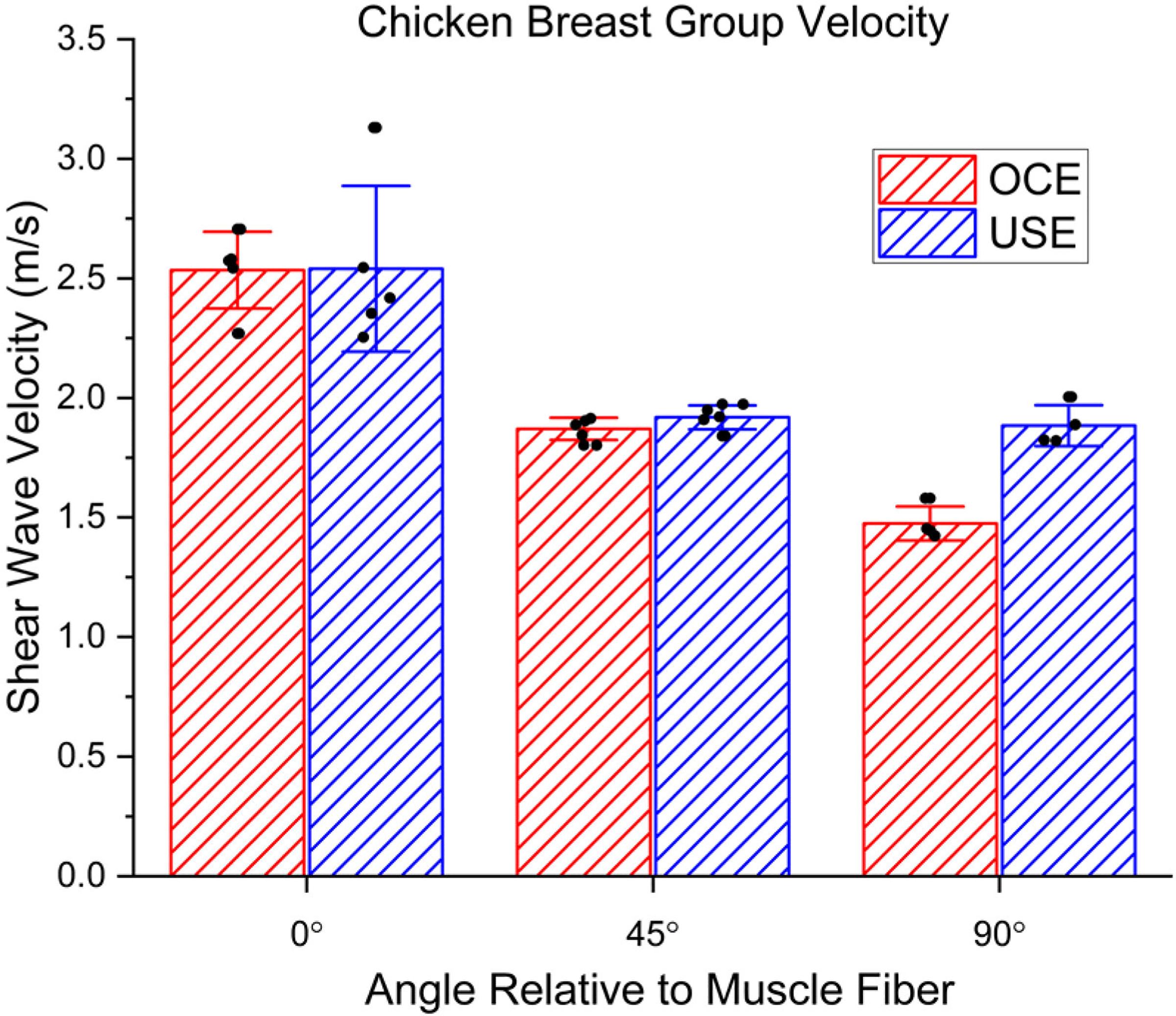
Comparison of USE and OCE shear wave group velocity measurements in chicken breast. The angles listed are with respect to the muscle fibers seen on the surface of the breast.

We then compared the ability of USE and OCE to resolve small changes in tissue stiffness, such as those that occur due to anisotropy. Both modalities can detect relatively small changes in elastic properties of homogeneous samples, as seen in the Bland-Altman analysis in Fig. 3. Because of the heterogeneous nature of the tissue [16] and the different operating depths, there will be differences between what is measured by USE and OCE. However, both techniques can differentiate between the different angles with respect to the muscle fiber orientation and were relatively consistent with each other as plotted in Fig. 4. The numerical results are shown in Table II. At 0° relative to the muscle fiber orientation, there was a difference between USE and OCE of 0.40%. At 45°, there was a difference of 2.6%. At 90°, there was a bigger separation, with a difference of 21%. Internal repeatability was analyzed using ICC (USE: 0.9385, OCE: 0.9924), which was excellent for both modalities.

## III. DISCUSSION

In general, OCE and USE are typically selected for very different situations by virtue of their strengths and limitations. USE is typically used to probe deeper, thicker tissues such as internal organs [17], [18], while OCE is used to gather information about surface-level or excised tissues due to its higher resolution but shallower imaging depth [19]–[21]. Though these use cases do not often overlap, the two modalities can be used interchangeably in cases where both are able to obtain good images, such as in skin [22], [23] or ocular tissues [20], [24]–[26].

Both modalities can detect small changes in elastic properties and have good intra-sample repeatability. ICC values were very high, indicating that measurements on the same sample were extremely repeatable and reliable during the phantom studies, as expected. Both typically differ from uniaxial testing by less than 15%.

Several clinical studies have included repeatability measures for both modalities and generally show that the methods are highly repeatable. Ultrasound shear wave elastography repeatability has been extensively studied with good results. A recent study for assessing liver fibrosis reported excellent repeatability with an ICC of 0.997 [27]. Another study using strain ultrasound elastography found that the technique had moderate to substantial repeatability as measured by ICC [28], while a group using shear wave elastography to monitor brain stiffness changes as a function of intracranial pressure reported repeatability of 92% as measured by ICC [29]. Additional studies have been done on other tissue types [30], [31], showing a high degree of repeatability. OCE, being a newer technique and only recently transitioning to clinical research, has fewer studies that focus on repeatability. OCE was used by one group to measure depth-dependent corneal displacements, and an ICC of 0.84 was reported [32]. Additionally, our group has previously published clinical work on OCE for the assessment of skin stiffness in systemic sclerosis patients and reported an ICC between continuous measurements of 0.93–0.98 and an ICC with a 5-minute break between measurements of 0.76–0.98 [33]. Additional clinical and non-clinical studies show a high degree of repeatability with multiple excitation methods [34]–[36].

We measured no significant bias nor change in bias as a function of elasticity. The limits of agreement for USE were 0.075 m/s, which was 2.26%. For OCE, the limits of agreement are 0.14 m/s, which was 3.99%. This means that if we were to measure a change in the elastic wave speed of 0.075 m/s (2.26%) using USE or 0.14 m/s (3.99%) using OCE, then we can say with 95% confidence that we measured something of different stiffness. The internal repeatability in the gelatin phantoms was excellent. Similarly, it was great in the chicken breast. While OCE and USE both tend to overestimate Young’s modulus, the estimations are reasonable and differ from uniaxial testing on average by 8.92% for OCE and 16.9% for USE. Overall, the results indicate that elastic moduli measured by USE and OCE are comparable, and the two modalities can be used interchangeably to measure tissue stiffness.

There are several limitations to this study. First, this paper does not control excitation frequencies. The air puff generated excitation bandwidths of 573 ± 13 Hz for 8% gelatin, 565 ± 15 Hz for 10%, 529 ± 25 Hz for 12%, and 501 ± 22 Hz for 14%, while USE produced excitation bandwidths of 1073 ± 150 Hz for 8%, 759 ± 95 Hz for 10%, 1112 ± 31 Hz for 12%, and 1186 ± 77 for 14%. When considering the effect dispersion has on measured velocities [37], [38], this could result in different measurements between OCE and USE. However, we would expect that OCE and USE would measure similar velocities in a given sample given similar excitation frequencies. Additionally, the two techniques use imaging modalities with vastly different imaging depths. Because of limited imaging depth, OCE measures Rayleigh waves at the surface of the sample instead of shear waves. The relationship between the two has been established, and we corrected for this when comparing speeds (See section V.F), but a direct comparison would undoubtedly be better.

OCE has several disadvantages when it comes to clinical imaging compared with USE. During OCE acquisition, several measurements are taken at points along a line, and repeated air pulses are required. This process takes significantly longer than USE, which can excite, image, and save the entire data set in a few seconds. In a clinical setting, these can be key constraints because the patient movement is often difficult to suppress and can result in measuring different locations than desired. This has been remedied by using ultra-fast framerate OCT systems, which are capable of performing OCE using only a single excitation as pioneered by our group or attaining an entire 4D data set (3D + time) in fractions of a second with high sweep rate lasers (>1 MHz) [39], [40].

However, OCE can measure changes on a time scale not possible with USE. OCE is limited by the scan rate of the laser, which allows for changes to be tracked at a higher effective frame rate. USE is limited by the speed of sound, though in essentially all clinical settings, physiological phenomena do not approach this limit. Additionally, OCE does not require coupling media provided that a suitable excitation source is selected. In USE, coupling media is required for ultrasound transmission and must be present even if an external excitation source is present. If ultrasound gel is used, air bubbles present within the gel can cause artifacts that may affect elastographic measurements [41].

Clinically, USE is much more widespread than OCE in part due to the fact that ultrasound imaging has been around since the late 1950s [42], while OCT was introduced in 1991 [3]. Recently, work has been focused on moving OCE toward the clinic in ophthalmology and dermatology due to its high spatial and temporal resolutions and ease of access to ocular tissues and skin. An active area of research involves customized crosslinking of corneal collagen to treat keratoconus [43]. With OCE, it is possible to generate high-resolution spatial maps before crosslinking to determine the weak areas of the cornea and after crosslinking to determine the effectiveness of the crosslinking technique [44]. Additionally, OCE has been able to obtain stiffnesses at the cellular scale, with a resolution better than 15 *μ*m [45]. It has also been used to assess myocardial infarction [46], to monitor fractional laser treatment of scar tissue [47], and as part of multimodal systems [11], [48].

It is important to note that some of the shortcomings of USE, such as its inability to measure very thin samples, can be overcome by choosing an external excitation method and using an imaging transducer with a much higher frequency. Axial resolution in ultrasound elastography, like in sonography, is determined by the ultrasound pulse width [49]. At very high frequencies, the resolution can be as low as 30 *μ*m [50], which begins to approach what is possible with optical imaging modalities such as OCT. At ultra-high frequencies (100–300 MHz), the resolution can reach ~6.2 *μ*m, which is comparable to optical imaging [7]. However, these transducers are not widely available and have only been fabricated in research laboratories. It has also been shown that different transducers, imaging depths, and imaging machines can have a statistically significant effect on the measured shear wave speed [51]. This is outside the scope of the current study but is the next step of this research.

## IV. CONCLUSION

This study directly compared OCE and USE for measuring elastic properties in homogeneous and heterogeneous samples. It also compared these results with the gold standard, which is uniaxial testing. Our results showed that OCE and USE are largely comparable, and either may be used provided that experimental constraints are considered. Future work will consider the effect excitation frequency and viscosity play when comparing OCE and USE and the effect of different transducers and depths on Young’s modulus values.

## V. MATERIALS AND METHODS

### A. PHANTOM CREATION

Eight gelatin phantoms were created by mixing porcine gelatin (gel strength 300, type A. Sigma-Aldrich Corp, MO, USA) with distilled water at concentrations of 8%, 10%, 12%, and 14% (w/w). Two cylindrical phantoms (D = 30 mm, H = 10 mm) of each concentration were created by pouring the mixture into standard culture dishes coated with petroleum jelly to prevent sticking and ease removal. Silica powder and black paint were added to the phantoms to create acoustic and optical scattering, respectively. The phantoms were removed from their molds and placed in weigh boats for imaging. A marking was placed on each weigh boat to denote the line across which OCE and USE measured to co-localize measurements.

### B. CHICKEN BREAST

Since many organs are heterogeneous [52]–[54] and/or anisotropic [55]–[57], the ability of these two techniques to reliably measure differences in tissue mechanical properties was also assessed. Chicken breast was roughly sliced into a cube with 3 cm sides. The muscle fiber orientation was noted by the surface evaluation of the fibers, and marks were made to ensure consistent measurements between USE and OCE: 0°, 45°, and 90° relative to the muscle fiber direction. Each location was measured five times.

### C. OCE MEASUREMENT

The OCE acquisition system has been described previously [58] and is shown in Fig. 1(left). In short, it consists of a broadband swept-source laser (HSL 2000, Santec Corp., Hackensack, NJ, USA) with a central wavelength of 1310 nm, scan range of 130 nm, scan rate of 30 kHz, axial resolution of 11 *μ*m in air, and transverse resolution of 16 *μ*m. A focused micro air pulse [58] was synchronized with the frame trigger during M-B-mode imaging [59] for an effective temporal resolution of 30 kHz. A wave was generated on the surface of the phantom by the air pulse and subsequently tracked at several points (N = 251) in a line (7.83 mm). The excitation was at the middle of the line. After each measurement, the sample was removed and replaced. Five trials were completed for each phantom.

### D. USE MEASUREMENT

The USE acquisition system consisted of a Vantage 256 (Verasonics, Kirkland, WA, USA) ultrasound system with a L11–5V transducer and is shown in Fig. 1(right). The imaging and push frequencies were 7.8 MHz and the pulse duration was 128 *μ*s. A thin layer of ultrasound gel was applied to the phantom surface. Special care was taken to ensure no bubbles were present. A shear wave was generated by the transducer at a focal point 4 mm below the surface and directly under the center of the transducer. Each push utilized 32 elements. After each measurement, the sample was removed and replaced to test repeatability. Five trials were completed for each phantom.

### E. UNIAXIAL COMPRESSION TESTING

The Young’s modulus of each phantom was measured via uniaxial mechanical compression testing (Model 5943, Instron Corp., Norwood, MA, USA). Each phantom was sliced into a rectangular prism approximately 10 mm in length by 10 mm in width by 18 mm in height prior to performing the measurements. Each phantom was coated with water to prevent friction from affecting the measurement. After contacting the phantom, the test began and continued until reaching 10% strain. Compression was performed at a rate of 0.25 mm/s. The phantom was removed and replaced after each measurement. Five trials were completed for each phantom.

### F. OCE ANALYSIS

OCE data were analyzed using custom MATLAB (Mathworks, Inc., Natick, MA, USA) software. At each spatiotemporal location, the displacement profile was averaged from the surface of the sample to ~400 *μ*m below the surface. The phase information was converted to displacement, and the surface motion and refractive index mismatch were corrected [60]. From this data, a spatiotemporal map was generated. This spatiotemporal map was used to determine group velocity by cross-correlation [61] followed by residual-weighted linear fitting. Fitting was performed on each side of the wave propagation and averaged for each repeated measurement.

Young’s modulus (YM) was calculated based on the surface wave group velocity (*C*_*g*_) using [62]:

$$E=\frac{2\rho {\left(1+v\right)}^{3}}{{\left(0.87+1.12v\right)}^{2}}C_{g}^{2},$$

where *ν* was Poisson’s ratio and *ρ* was the mass density of the medium. We assumed *ν* = 0.5 and *ρ* = 1000 kg/m^3^.

The Rayleigh waves in the incompressible medium have a speed that is ~95.5% of the shear wave speed [20]. These waves are what is measured by OCE, and the speeds were adjusted accordingly for direct comparison with USE in Figs. 3 and 4.

### G. USE ANALYSIS

USE data were analyzed using custom MATLAB software. The displacement at each spatial location within the image at each time was calculated using Loupas’ algorithm and was used for further analysis [63]. Directional filtering was performed in order to isolate left-moving and right-moving waves [64]. Each propagation direction was analyzed separately. A 400 *μ*m region near the push focus was averaged in depth to improve SNR and used to create a spatiotemporal map. Cross-correlation was then performed, followed by residual-weighted linear fitting to determine the velocity. The velocity from each side was averaged for each measurement.

Young’s modulus was calculated based on the shear wave group velocity *c* using [65]:

$$E=3\rho c^{2},$$

where *ρ* = 1000 kg/m^3^ was the mass density of gelatin.

### H. BLAND-ALTMAN ANALYSIS

Bland-Altman analysis was performed on each phantom [14]. For a given phantom, the velocity was calculated for each of the five trials. The mean of these five trials was plotted on the x-axis. The difference from the mean for each trial was plotted on the y-axis. The average of the differences was calculated as the bias. The mean ±1.96 standard deviations of the differences were calculated as the upper and lower limits of agreement. The data was normalized by dividing the differences of each trial by the mean of the five trials for each sample. This was repeated for each phantom.

## Figures and Tables

**TABLE I.** Average Young’s Modulus Values in Kpa for Each Gelatin Concentration

	Young’s Modulus (kPa)		
**Conc.**	OCE	USE	UT
**8%**	22.84 ± 1.90	24.10 ± 1.55	21.63 ± 1.28
**10%**	30.74 ± 2.0	35.36 ± 0.66	26.12 ± 1.1
**12%**	38.39 ± 2.1	45.74 ± 1.2	38.89 ± 0.16
**14%**	63.08 ± 5.9	58.57 ± 2.7	56.77 ± 1.2

**TABLE II.** Chicken Breast Group Velocity Vs Angle Relative to Muscle fiber. Angles Ranged From 0° (along Muscle fiber) to 90° (perpendicular to Muscle fiber)

Angle relative to fiber orientation	USE velocity (m/s)	OCE velocity (m/s)
**0°**	2.54 ± 0.35	2.53 ± 0.16
**45°**	1.92 ± 0.05	1.87 ± 0.05
**90°**	1.87 ± 0.08	1.47 ± 0.07

## IMPACT STATEMENT

USE and OCE can be used interchangeably in select applications provided that certain constraints are considered. This allows clinicians and researchers greater freedom to choose the appropriate modality.
